# Anticancer Properties of *Plectranthus ornatus*-Derived Phytochemicals Inducing Apoptosis via Mitochondrial Pathway

**DOI:** 10.3390/ijms231911653

**Published:** 2022-10-01

**Authors:** Przemysław Sitarek, Ewelina Synowiec, Tomasz Kowalczyk, Gabrielle Bangay, Tomasz Śliwiński, Laurent Picot, Salvatore Princiotto, Patricia Rijo

**Affiliations:** 1 Department of Biology and Pharmaceutical Botany, Medical University of Lodz, ul. Muszyńskiego 1, 90-151 Lodz, Poland; 2 Laboratory of Medical Genetics, University of Lodz, Pomorska 141/143, 90-236 Lodz, Poland; 3 Department of Molecular Biotechnology and Genetics, University of Lodz, 90-237 Lodz, Poland; 4 CBIOS—Universidade Lusófona’s Research Center for Biosciences and Health Technologies, 1749-024 Lisbon, Portugal or; 5 Littoral Environnement et Sociétés UMRi CNRS 7266 LIENSs, La Rochelle Université, 17042 La Rochelle, France; 6 Instituto de Investigação do Medicamento (iMed.ULisboa), Faculdade de Farmácia, Universidade de Lisboa, 1649-003 Lisbon, Portugal

**Keywords:** *Plectranthus ornatus*, anticancer effect, apoptosis induction, diterpenes, mitochondrial pathway

## Abstract

Since cancer treatment by radio- and chemotherapy has been linked to safety concerns, there is a need for new and alternative anticancer drugs; as such, compounds isolated from plants represent promising candidates. The current study investigates the anticancer features of halimane (11R*,13E)-11-acetoxyhalima-5,13-dien-15-oic acid (HAL) and the labdane diterpenes 1α,6β-diacetoxy-8α,13R*-epoxy-14-labden-11-one (PLEC) and forskolin-like 1:1 mixture of 1,6-di-*O*-acetylforskolin and 1,6-di-*O*-acetyl-9-deoxyforskolin (MRC) isolated from *Plectranthus ornatus* in MCF7 and FaDu cancer cell lines. Cytotoxicity was assessed by MTT assay, ROS production by Di-chloro-dihydro-fluorescein diacetate assay (DCFH) or Red Mitochondrial Superoxide Indicator (MitoSOX) and Mitochondrial Membrane Potential (MMP) by fluorescent probe JC-1 (5′,6,6′-tetrachloro-1,1′,3,3′-tetraethylbenzimidazolylcarbocyanine iodide). In addition, the relative amounts of mitochondrial DNA (mtDNA) were determined using quantitative Real-Time-PCR (qRT-PCR) and damage to mitochondrial DNA (mtDNA) and nuclear DNA (nDNA) by semi-long run quantitative Real-Time-PCR (SLR-qRT-PCR). Gene expression was determined using Reverse-Transcription-qPCR. Caspase-3/7 activity by fluorescence was assessed. Assessment of General In Vivo Toxicity has been determined by Brine Shrimp Lethality Bioassay. The studied HAL and PLEC were found to have a cytotoxic effect in MCF7 with IC_50_ = 13.61 µg/mL and IC_50_ = 17.49 µg/mL and in FaDu with IC_50_ = 15.12 µg/mL and IC_50_ = 32.66 µg/mL cancer cell lines. In the two tested cancer cell lines, the phytochemicals increased ROS production and mitochondrial damage in the *ND1* and *ND5* gene regions and reduced MMP (ΔΨm) and mitochondrial copy numbers. They also changed the expression of pro- and anti-apoptotic genes (*Bax*, *Bcl-2*, *TP53*, *Cas-3*, *Cas-8*, *Cas-9*, *Apaf-1* and *MCL-1*). Studies demonstrated increase in caspase 3/7 activity in tested cancer cell lines. In addition, we showed no toxic effect in in vivo test for the compounds tested. The potential mechanism of action may have been associated with the induction of apoptosis in MCF7 and FaDu cancer cells via the mitochondrial pathway; however, further in vivo research is needed to understand the mechanisms of action and potential of these compounds.

## 1. Introduction

Cancer remains the leading cause of death worldwide. Given the morbidity and mortality associated with this disease, as well as the significant economic burden, there is still an urgent need to develop more effective treatment strategies [1,2,3]. The disease affects people in both developing and developed countries and its overall incidence is further compounded by gradual aging of the population. An important approach to alleviating this enormous burden on public health is cancer chemoprevention, the use of synthetic or natural agents to inhibit, delay, or reverse carcinogenesis. Plants produce diverse compounds, including secondary metabolites, that have been used as antibiotics, painkillers, anticancer and cardioprotective drugs for over 5000 years. This enormous potential of plant compounds, which has today been further refined by the development of science and the ability to accurately identify plant-based bioactive compounds, has fostered the development of new health-related technologies [4,5,6]. Research into the anticancer properties of many plants is generating very promising results. About 80–90% of the population in developing countries still uses drugs based on plant extracts. The isolation and identification of biologically-active compounds of plant origin has led to the discovery of new therapeutic agents, stimulating the development of the health and pharmaceutical sectors [7,8,9,10,11].

Many of these compounds, such as vinblastine (VBL) and vincristine (VCR), etoposide, paclitaxel (Taxol^®^, Bristol-Myers Squibb, New York, USA), docetaxel, topotecan and irinotecan, have been successfully used in anti-cancer therapy [4,12]. Currently, new phytochemicals are sought, which can act as sources of new molecules for the development of new, innovative drugs for many branches of medicine, including Oncology. One interesting plant with a multidirectional action and strong biological properties is the genus *Plectranthus*, named after the Latin *plecton* meaning spur and *anthos* for flower, first described in 1788 by the French botanist Charles L’Héritier. In Africa and Asia, *Plectranthus* is mostly used for digestion, pain, skin ailments and infections, whereas Caribbean populations have exploited it for epilepsy symptom relief [13,14]. Our earlier studies confirm that diterpenes (Roy, Deroy, Diroy, Parv) isolated from *Plectranthus* sp. induced apoptosis in a number of cancer cell lines through various signaling pathways [15,16,17].

The secondary metabolites discovered in this genus have been classified as monoterpenoids, sesquiterpenoids, diterpenoids and phenols, with the most diverse diterpenoids being abietane diterpenoids. The labdane diterpenoid, forskolin, is found in *Plectranthus barbatus* and may explain some of its traditional uses [13]. Forskolin compounds are known to have several biological activities regarding their cardiotonic, antiplatelet anti-inflammatory and anticancer properties. Concerning their anticancer activity, recent studies have demonstrated the potential of forskolin in effective cancer therapeutics in U937 acute myeloid leukemia cell lines, specifically by enhancing the antiproliferative effects of a small molecule inhibitor, GSKJ4, by apoptotic cell death, as well as down-regulation of the BCL2 protein, caspase 3 activation and PARP protein cleavage. The authors propose that the presence of forskolin sensitizes the leukemia cells to the small molecule inhibitor, GSKJ4, via a cAMP/PKA-mediated pathway. Similarly, forskolin was shown to potentiate the antiproliferative effects of doxorubicin in triple negative breast cancer and sensitize the MDA-MB-231 and MDA-MB-46 cell lines to doxorubicin [18,19,20,21]. Another labdane diterpene is Plectronatin C (PLEC), isolated from *Plectranthus ornatus*. Earlier studies have shown this compound to have moderate antifungal activity and to inhibit cyclooxygenase-2 (COX-2) [22,23]. In turn, diterpenes with a haliman skeleton (HAL) still have few pharmacological properties. In 2011, it was reported that some haliman derivatives derived from haliman A diterpenes, namely haliman B-D diterpenes, are potent COX-2 inhibitors [22].

*Plectranthus ornatus*-isolated secondary metabolites such as HAL, PLEC and MRC clearly have promising ethnopharmacology and bioactivity value. Therefore, the aim of this work was to determine the biological activity of these diterpenes with regard to their potential anti-cancer value.

## 2. Results

### 2.1. Cytotoxicity

**Figure 1:**
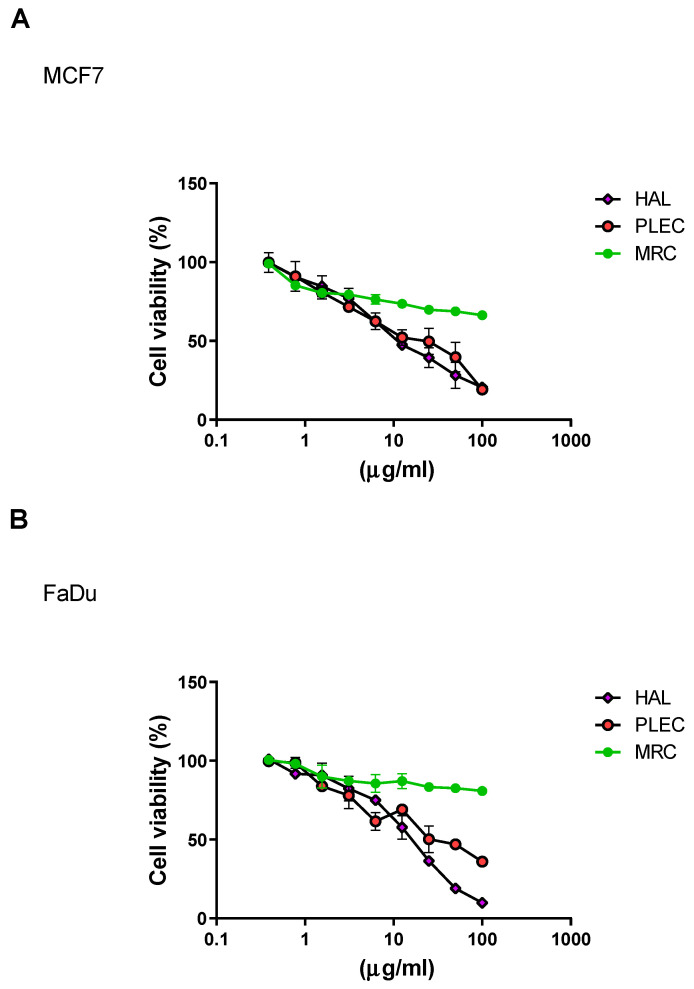
Effects of HAL, PLEC and MRC on the viability of (**A**) MCF7, (**B**) FaDu cells, as determined by the MTT assay. Values represent the means ± SD as percent (%) of control values. Values on the *X*-axis are shown as log10.

The cytotoxic effects of HAL, PLEC and MRC treatment against the MCF7 and FaDu cell lines is given in Figure 1A,B. For the MCF7 cell line, HAL and PLEC had cytotoxic effects, with IC_50_ values of 13.61 µg/mL and 17.49 µg/mL, respectively. Similarly, HAL (IC_50_ = 15.12 µg/mL) and PLEC (IC_50_ = 32.66 µg/mL) demonstrated cytotoxic activity against the FaDu cell line. In turn, the MRC showed a moderate cytotoxic effect for both tested cell lines (stronger for the MCF7 cell line). The IC_50_ concentration for HAL and PLEC was used for further studies, while the highest tested concentration was used for MRC.

### 2.2. Reactive Oxygen Species (ROS) Generation

**Figure 2:**
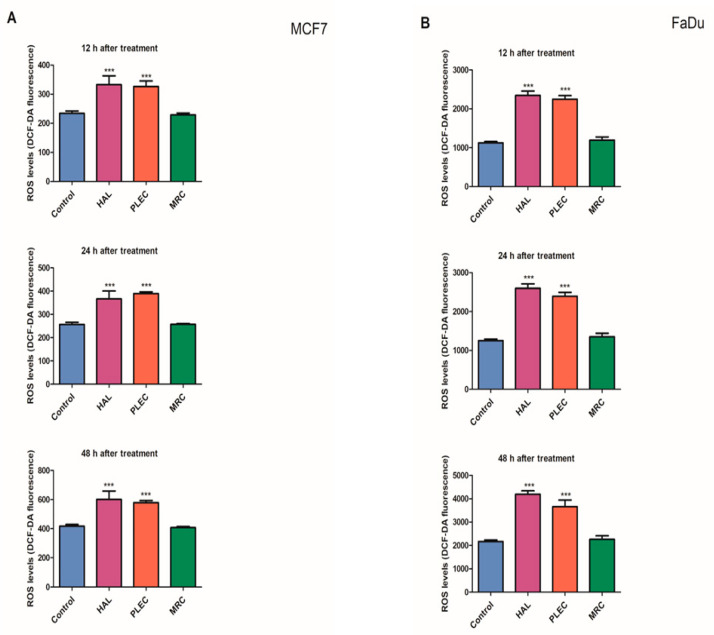
(**A**): Formation of Reactive Oxygen Species (ROS) in MCF7 cells treated with HAL, PLEC and MRC for 12, 24 and 48 h. Results are given as means ± SD. *** *p* < 0.001, significant differences between treated and untreated (control) cells. (**B**): Formation of Reactive Oxygen Species (ROS) in FaDu cells treated with HAL, PLEC and MRC for 12, 24 and 48 h. Results are given as means ± SD. *** *p* < 0.001, significant differences between treated and untreated (control) cells.

The ROS level in the MCF7 and FaDu cells was also measured after 12, 24 and 48 h treatment with HAL, PLEC and MRC (Figure 2A,B). After 12 h incubation of MCF7 cells with HAL and PLEC, the ROS level was significantly higher (*p* < 0.001) than controls and this effect was maintained for up to 48 h. In the FaDu cell line, a significant increase in ROS level was noted after 12 h of incubation with HAL and PLEC and that level was maintained up until 48 h. MRC treatment did not result in any change in ROS level over the studied time period for either cell line.

### 2.3. Mitochondrial ROS Generation

**Figure 3:**
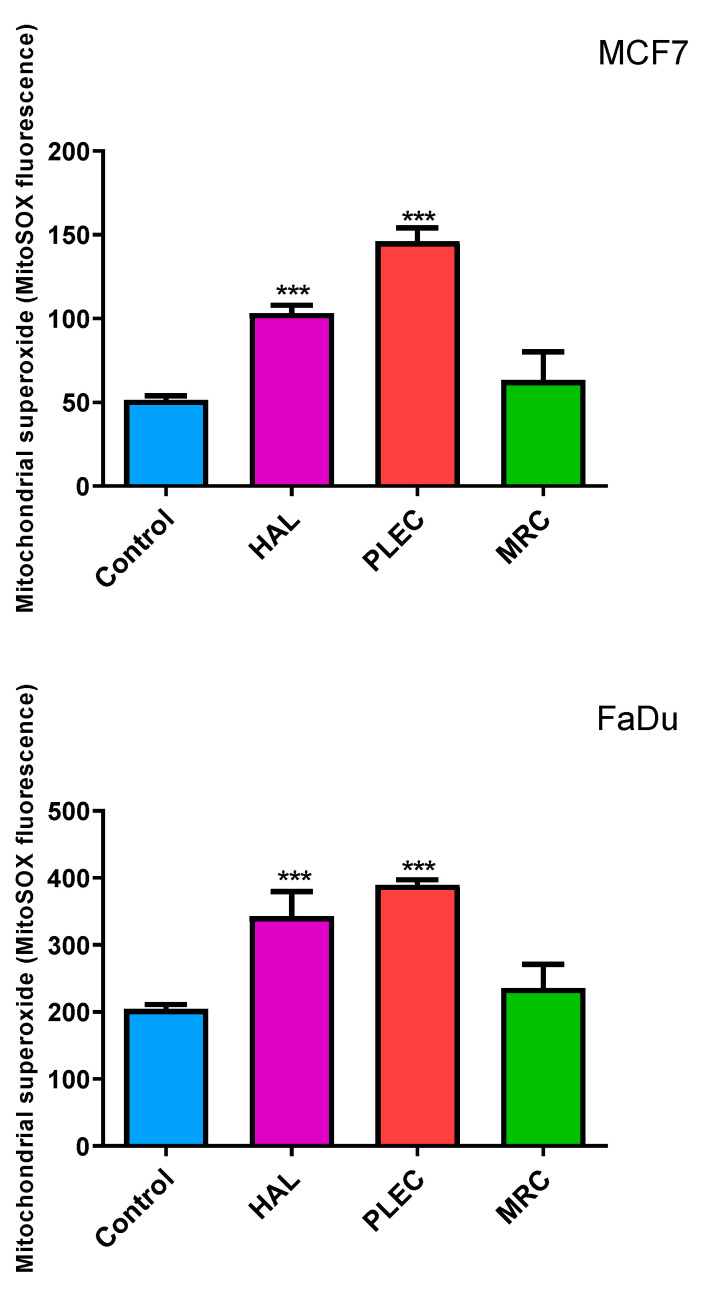
Generation of mitochondrial superoxide in MCF7 and FaDu cells as detected with MitoSOX. The cells were exposed to HAL, PLEC and MRC and, after 24 h of incubation, MitoSOX Red fluorescence was measured using a microplate reader. Data are means ± SD (n = 3). *** (*p* < 0.001) as compared with control (untreated) cells.

Our study showed an increase in mitochondrial superoxide (referred to as mitochondrial ROS, or mROS) levels for both FaDu and MCF7 cancer cell lines tested after incubation with HAL and PLEC compounds. No statistically significant changes were shown for the MRC compound (Figure 3).

### 2.4. Mitochondrial Membrane Potential

**Figure 4:**
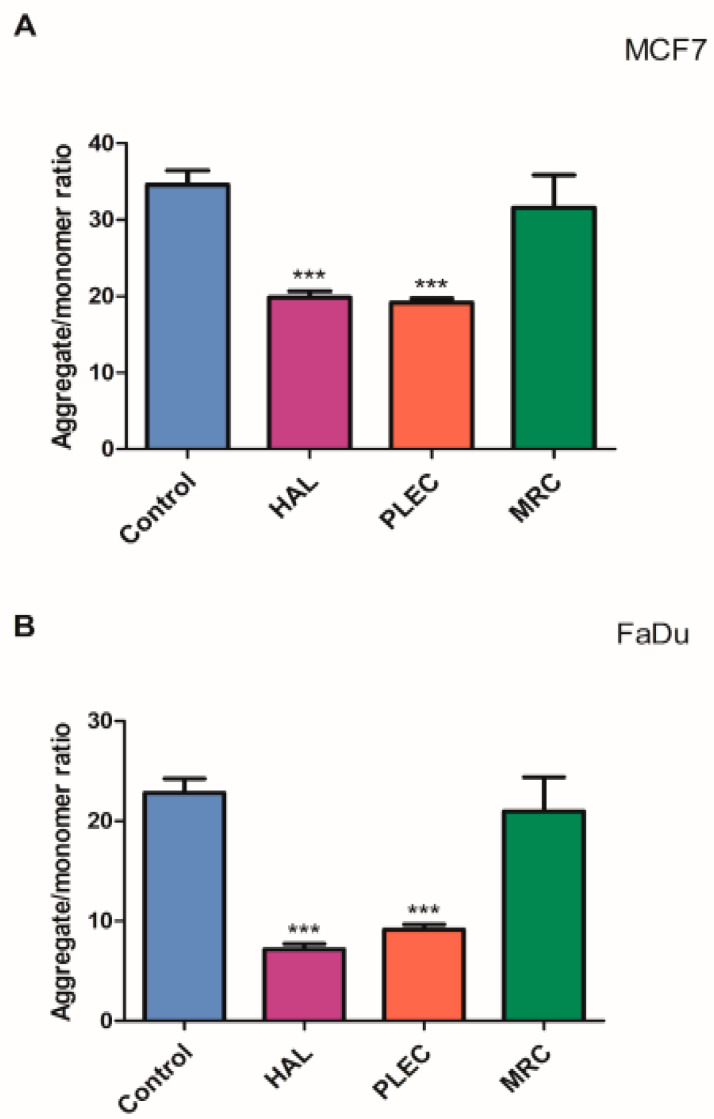
Changes in mitochondrial membrane potential of (**A**) MCF7, (**B**) FaDu cells incubated with HAL, PLEC and MRC for 24 h. Results are presented as means ± SD. *** statistically significant changes compared with control cells (*p* < 0.001).

The mitochondrial membrane potential (MMP) in the MCF7 cell line and FaDu cell lines incubated with HAL, PLEC and MRC for 24 h are given in Figure 4A,B. In both lines, a statistically significant reduction in MMP was observed after treatment with HAL and PLEC, but not for MRC.

### 2.5. Mitochondrial Copy Number

**Figure 5:**
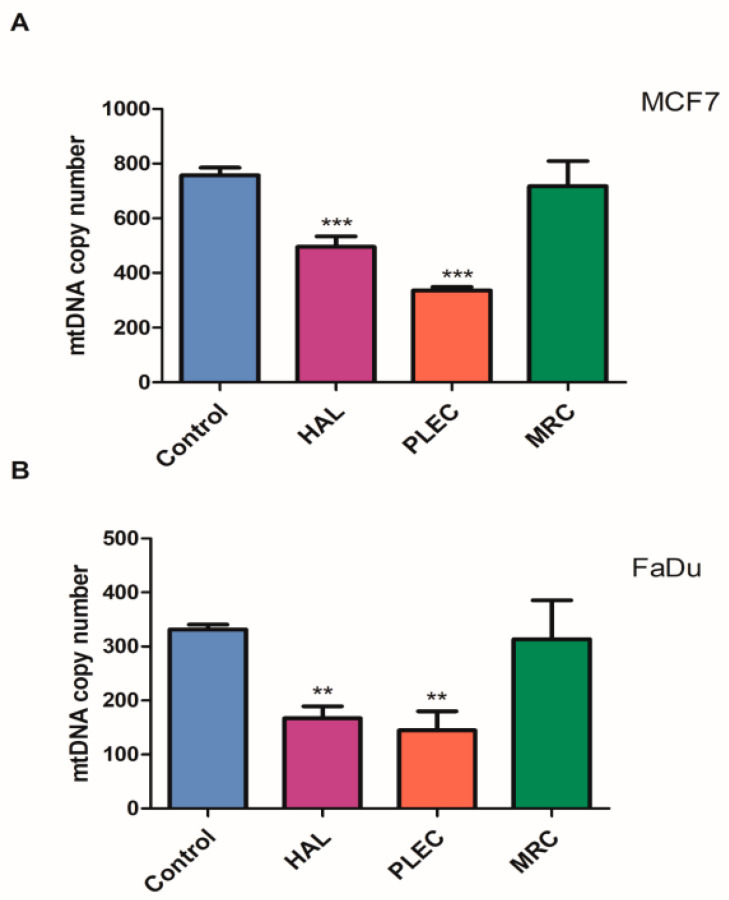
(**A**,**B**). Mitochondrial DNA copy number in MCF7 (**A**) and FaDu (**B**) cells measured by real-time quantitative PCR. The cells were exposed to HAL, PLEC and MRC and, after 24 h of incubation, DNA was extracted. Values are expressed as the means ± SD. ** *p* < 0.01, *** *p* < 0.001 vs. control group.

Additionally, 24 h of incubation with HAL and PLEC resulted in a lower mtDNA copy number in both the MCF7 and FaDu cells compared to controls. However, no statistically significant differences in mtDNA copy number were found for the cells treated with MRC (Figure 5A,B).

### 2.6. Quantification of Mitochondrial DNA (mtDNA) Damage

**Figure 6:**
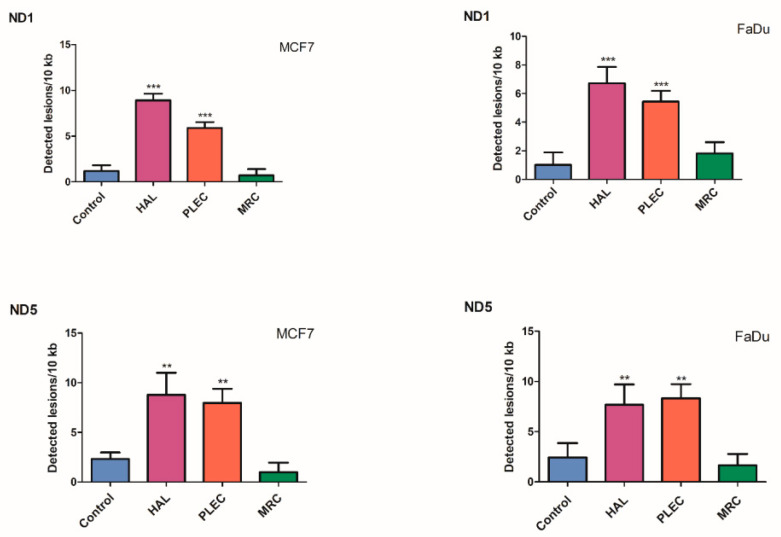
Quantification of mitochondrial DNA (mtDNA) lesion frequency per 10 kb DNA by SLR-qRT-PCR amplification of total DNA from MCF7 and FaDu cells exposed to HAL, PLEC and MRC for 24 h. Data represents the mean ± SD of three replicates. ** *p* < 0.01; *** *p* < 0.001 vs. control group.

The results of mtDNA damage analysis resulting from 24 h HAL, PLEC and MRC exposure is given in Figure 6. In the FaDu cell line, a significant (*p* < 0.001) increase in lesion rate was noted in the ND1 and ND5 regions: 6.72 (ND1) and 7.67 (ND5) lesions per 10 kb DNA treated with HAL, 5.43 (ND1) and 8.33 (ND5) lesions per 10 kb DNA after PLEC treatment. In the MCF7 cells, HAL treatment exhibited higher mtDNA damage (8.89 lesions per 10 kb DNA in ND1 and 8.78 lesions per 10 kb DNA in ND5) as did PLEC, being 5.88 for the ND1 region and 7.95 for the ND5 region. MRC treatment did not influence the level of mtDNA damage.

### 2.7. Quantification of Nuclear DNA (nDNA) Damage

**Figure 7:**
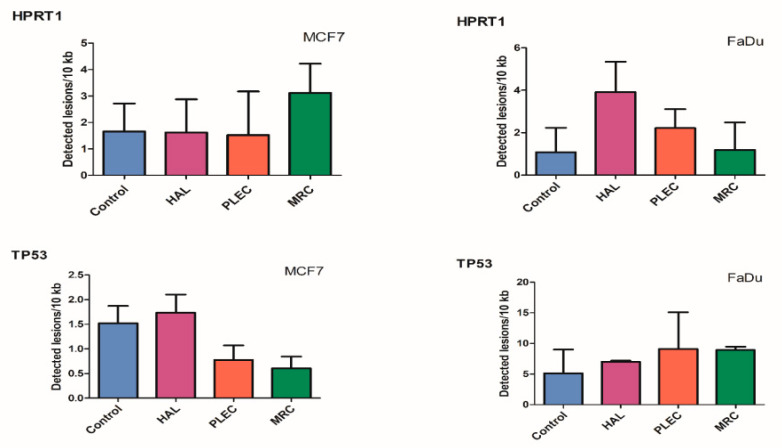
Quantification of nuclear DNA (nDNA) lesion frequency per 10 kb DNA by SLR-qRT-PCR amplification of total DNA from MCF7 and FaDu cells exposed to HAL, PLEC and MRC for 24 h. Data represents the mean ± SD of three replicates.

No significant difference in the level of nDNA damage in the HPRT1 and TP53 regions was found between treated cells and control MCF7 and FaDu cells (Figure 7).

### 2.8. Changes in Gene Expression

**Figure 8:**
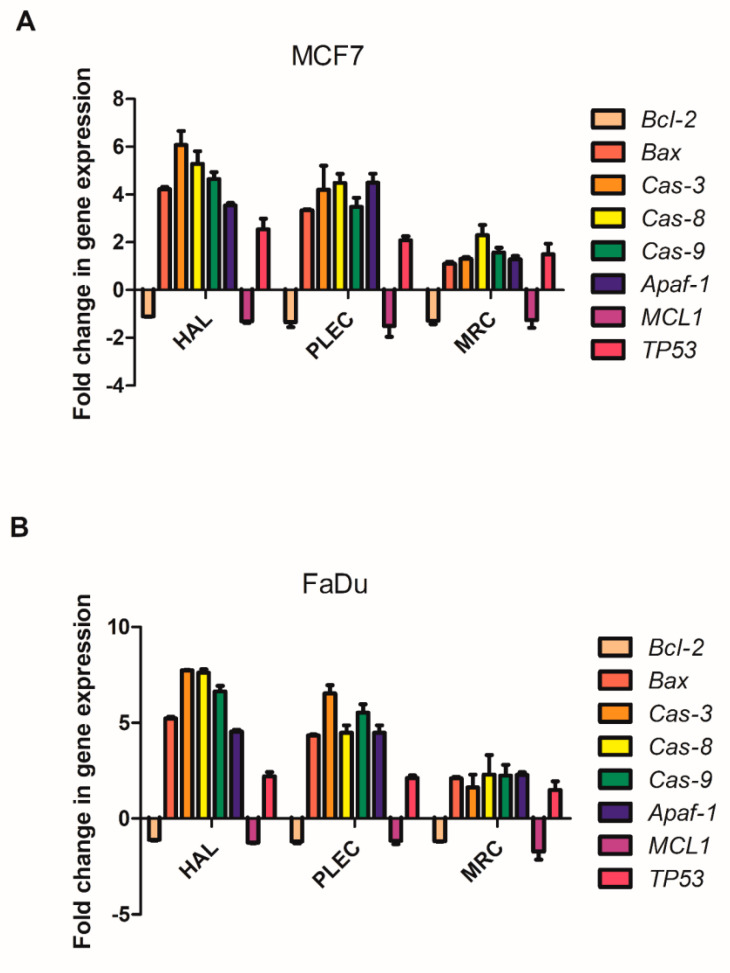
(**A**,**B**)**.** Gene expression (*Bax, Bcl-2 Cas-3, Cas-8, Cas-9, Apf-1 MCL1* and *TP53*) for MCF7 (panel **A**) and FaDu (panel **B**) cell lines after 24 h treatment with tested compounds. Data is presented as fold change in cells treated with HAL, PLEC and MRC compared to untreated cells, in which the expression level was taken as 1. The mean values ± SD (n = 3).

The changes in *Bcl-2, Bax, Cas-3, Cas-8, Cas-9, Apaf-1, MCL1* and *TP53* gene expression after treatment with HAL, PLEC and MRC in MCF7 and FaDu cell are given in Figure 8A,B. For both tested lines, HAL and PLEC increased *Bax, Cas-3,8,9, Apaf-1* and *TP53* mRNA level. Only *Bcl-2* and *MCL1* expression decreased in both lines.

### 2.9. Induction of Apoptosis

**Figure 9:**
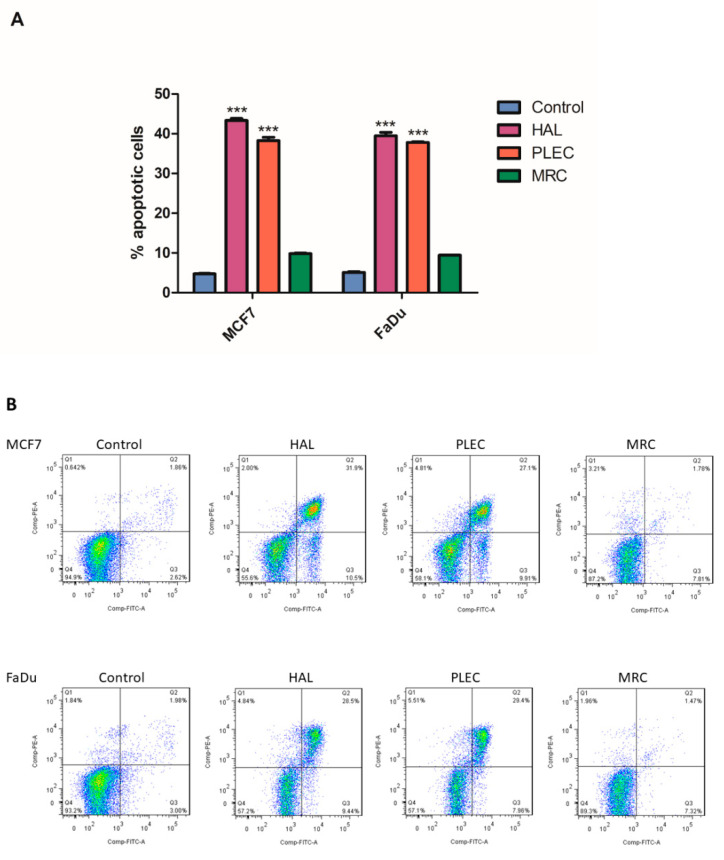
The effect of HAL, PLEC and MRC on apoptosis induction in MCF7 and FaDu cancer cells. Values are means ± SD (n = 3). *** *p* < 0.001 vs. control group (upper panel **A**). The graph shows % apoptotic cells as the sum of cells in early and late apoptosis. The dot plots show (lower panel **B**) results of one representative experiment for each kind of cells treated with HAL, PLEC, MRC. Q2 and Q3 quadrant presents a fraction of late and early apoptotic cells, respectively.

Significantly higher % apoptotic cell values were observed in MCF7 and FaDu after treatment with HAL and PLEC compared to controls. However, no change was shown for MRC (Figure 9).

### 2.10. Induction of Apoptosis (Caspase-3/7 Levels)

**Figure 10:**
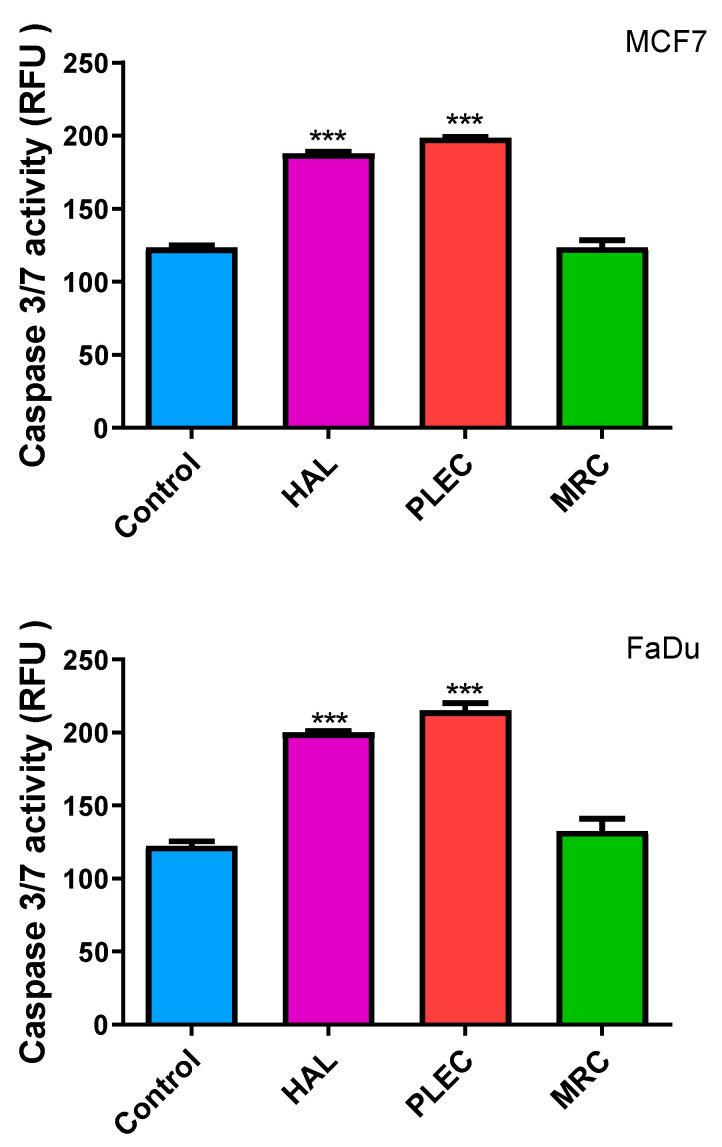
Action of HAL, PLEC and MRC on Caspase-3/-7 activity levels. MCF7 and FaDu cells were treated with appropriate compounds for 24 h, stained with 5 μM CellEvent™ Caspase-3/-7 Green detection reagent for 30 min at 37 °C and fluorescence immediately measured. Data represents the mean ± SD of three replicates. *** (*p* < 0.001) vs. untreated (control) cells.

To test whether HAL, PLEC and MRC isolated from *Plectranthus ornatus* induced apoptosis, we examined caspase 3/7 activity in FaDu and MCF7 cancer cells treated for 24 h. Two tested compounds HAL and PLEC showed significant effects on caspase 3/7 activation, while no statistically significant changes were shown for the compound MRC (Figure 10).

### 2.11. General Toxicity in In Vivo Test

**Figure 11:**
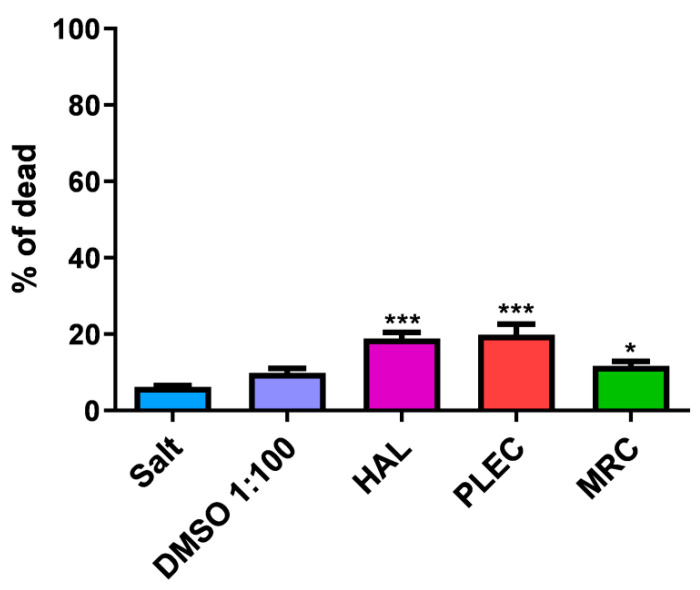
Mortality rate (%) of Artemia salina after 24 h exposure to HAL, PLEC and MRC from *P. ornatus.* Values are expressed as the mean ± SD (n = 3). * *p* < 0.05; *** *p* < 0.001 comparison salt vs. HAL, PLEC and MRC.

Our study showed no toxic effect in the Brine shrimp test for all compounds (HAL, PLEC and MRC) at the tested concentration (100 µg/mL) after 24 h incubation (Figure 11).

## 3. Discussion

The plant kingdom is an important source of new pharmacologically active molecules, providing a library of promising therapeutic agents that can be used in many branches of medicine [24]. A huge number of popular drugs are derived directly or indirectly from natural products, and many remain in clinical use, including in Oncology, such as paclitaxel, vinblastine, vincristine and podophyllotoxin [4,25,26,27]. Despite the steadily growing popularity of synthetic products, which has led to the many new drugs that exist today, plant secondary metabolites remain key to drug design, as their basic structures serve as templates for the synthesis or semi-synthesis of new substances to treat diseases [28,29]. Although the results of reports on the safety, efficacy and use of synthetic drugs remain a matter of debate in the medical community, naturally occurring bioactive molecules of plant origin still play a dominant role in the treatment of ailments due to their high tolerance and acceptance by patients during treatment. The use of typical synthetic drugs in chemotherapy is associated with serious side effects such as decreased appetite, hair loss, gastrointestinal changes, neurological dysfunction, and drug resistance, which weaken the patient and reduce the quality of life, both mentally and physically [16,24,26,28,30,31,32,33]. Studies have shown phytochemicals to be less toxic and more effective, in some regards. Hence, there is a great need to search the plant world for new compounds with potential medical effects [28,34,35,36,37]. An interesting source of secondary metabolites is the genus *Plectranthus* belonging to the Lamiaceae family. This genus comprises about 300 species distributed in Tropical Africa, Asia and Australia and some species are well documented for their ethnomedicinal uses [38]. Numerous studies have shown the content of various classes of compounds and the molecules isolated from them have shown many interesting biological properties. Cretton et al. described 11 terpenoids isolated from *P. scutellarioides,* among which six compounds are previously undescribed phytochemicals, while investigating their cytotoxic effects in human multiple myeloma stem cells (MM-CSCs) and multiple myeloma plasma cells RPMI 8226 [38]. Our previous studies showed that abietane diterpenes can induce apoptosis in many types of cancer cells through a variety of signaling pathways [17,39,40,41]. These promising results prompted a further search for other active compounds of the *Plectranthus* genus with potentially medical applications, particularly among the diterpenes.

Diterpenoid compounds are a large family of natural products whose biological activity is enhanced by the presence of a lactone group. The diterpenoids include labdane, the halimans and clerodans. These compounds have a decalin system with two fused rings (A and B) and a side chain at position 9 [42,43]. In turn, biosynthetically, halimans and clerodans appear to be related to labdans through a series of methyl and hydride shifts. The halimans have an intermediate carbon skeleton between the labdan and clerodan skeletons. Some of these compounds have been found to possess antitumor, antimicrobial and mosquito repellent properties and to inhibit germination, and act as biomarkers for tuberculosis; however, many of the halimane diterpenoids have not been biologically evaluated [42,43,44,45].

This work examines the biological properties of the halimane compound HAL, PLEC and MRC isolated from the *Plectranthus ornatus* plant.

The MTT assay results confirm the cytotoxic potency of HAL and PLEC in treated MCF7 and FaDu cancer cells in the tested range concentrations. IC_50_ for HAL was 13.61 µg/mL and for PLEC 17.49 µg/mL in MCF7 cells. In turn, for HAL this was 15.12 µg/mL and for PLEC 32.66 µg/mL in FaDu cells, respectively. Weaker cytotoxic effect was demonstrated for labdane diterpenes (forskolin-like 1:1 mixture of 1,6-di-*O*-acetylforskolin and 1,6-di-*O*-acetyl-9-deoxyforskolin) MRC compound in the concentration range tested. A previous study reported no cytotoxicity up to 25 μg/mL on normal PBMC-derived macrophages [46]. Our findings are the first to confirm that these compounds have cytotoxic effects against MCF7 and FaDu cancer cell lines. Cretton et al. demonstrated that the abietane diterpene Coleon O also exhibited significant activity towards human multiple myeloma cancer stem cells and RPMI 8226 cells, with IC_50_ values of 9.2 and 8.4 μM, respectively [38]. In addition, our study showed no toxic effect in an in vivo test on brine shrimp after 24 h of incubation.

The study also investigated the potential apoptotic mechanism of action. Apoptosis is a very tightly controlled process that regulates the development and homeostasis of organisms. Specific symptoms of this process are a reduction in cell volume, thickening of cytoplasmic organelles, condensation and fragmentation of nuclear chromatin and activation of the cysteine protease caspase family [47,48]. A characteristic feature, among others, is that the extrinsic death receptor pathway involves the activation of caspase-8, which then cleaves caspase-3, leading to apoptosis [49,50]. On the other hand, the intrinsic, mitochondrial pathway is regulated by Bcl-2 family proteins, including the anti- and proapoptotic factors (Bcl-2 and Bax) and involves the release of cytochrome c (Cytc) from the mitochondria, followed by the arrangement of a complex containing procaspase-9, cytochrome c and protease activation factor (Apaf)-1. The released Cytc then activates caspase-9, which later induces caspase-3 activation, leading to apoptosis [51,52,53,54]. Our results demonstrate that HAL, PLEC can induce apoptosis via mitochondrial accumulation of ROS, which may induce mitochondrial damage by altering mitochondrial membrane potential, changing the number of mitochondrial copies and increasing mitochondrial DNA damage in the ND1 and ND5 gene regions. Our findings indicate increased ROS production in MCF7 and FaDu cells after treatment with HAL and PLEC but no such result was observed for MRC. ROS accumulation is known to be associated with mitochondrial damage, which induces apoptosis. The destructive function of ROS release (RIRR) may have a physiological role that involves the elimination of unwanted cells. Adaptive release of sufficient ROS in the vicinity of mitochondria may lead to activation of local pools of enzymes involved in protective signaling pathways [55,56]. An increase in ROS can cause damage to the mitochondria, which has also been confirmed in our studies. The multivariate analysis showed a decrease in the mitochondrial membrane potential, a change in the number of mitochondrial copies and an increase in mitochondrial damage in the ND1 and ND5 regions of the genes in both tested MCF7 and FaDu cancer cell lines. However, no changes in nuclear damage were observed in the regions of the HPRT1 and TP53 genes. Mei et al. demonstrated that reduced mtDNA copy number can elevate ROS levels in cancer cells and increases the sensitivity of these cells to chemotherapeutic drugs used and the rate of apoptosis [57]. In contrast, Sun et al. noted that increased mitochondrial DNA copy number, caused by forced expression of mitochondrial transcription factor A, significantly accelerates cell proliferation and has an inhibitory effect on apoptosis of microsatellite-stable colon cancer cells [58]. Indeed, abietane diterpene treatment has been found to reduce mitochondrial copy numbers in MCF7 cancer cells, which is in line with our current results after treatment with halimane and labdane diterpenes [17]. The next step in confirming apoptosis by the mitochondrial pathway is the presence of changes in the expression of pro and antiapoptotic genes. It has been reported that Bcl-2 and Bax play an important role in causing apoptosis through the mitochondrial pathway. The Bcl-2 protein is an antiapoptotic molecule and its increased expression delays the disruption of organelles in apoptotic cells. In addition, activation of the caspase cascade may induce apoptosis [59,60]. We showed a change in the expression level of genes directly related to the apoptotic pathway, such as *Bax, Bcl-2, TP53, Cas-3, Cas-8, Cas-9, Apaf-1* and *MCL-1*, which confirms our hypothesis that HAL and PLEC induce apoptosis via the mitochondrial pathway in MCF7 and FaDu cancer cells. In addition, we showed an increase in caspase-3/7 activity for the same compounds. This is the first such report on the effect of these compounds on the induction of apoptosis in MCF7 and FaDu cancer cells.

The MRC compound did not show significant changes in the expression levels of the genes tested, which may indicate its low biological activity in this study area. It is difficult for us, at this stage of the study, to determine what the poor activity of MRC is due to. We suspect that it may be due to the mixture of the two compounds in its formulation, or to the chemical structure or different sensitivity of the individual lines resulting from their origin. Therefore, further research is important to find out other properties of this compound.

Moreover, Annexin-V FITC analysis confirmed that treatment with the tested compounds induced apoptosis in both the MCF7 and FaDu cells. Annexin-V FITC detects apoptosis by targeting for binding to negatively charged phosphatidylserine (PS) at the outer plasma membrane [61]. Hence, staining with annexin V/PI showed that HAL and PLEC in IC_50_ concentration increase the rate of apoptosis in the MCF7 and FaDu cells; however, this was not observed for MRC. This is the first confirmation that halimane and labdane diterpenes influence apoptosis induction in MCF7 and FaDu cancer cells. Our previous study revealed that abietane diterpenes such as: Diroy, Parv, Deroy and Roy isolated from *Plectranthus madagascariensis* and *Plectranthus ecklonii* induced apoptosis in human leukemia cell line by mitochondrial pathway [17]. In turn, the same compounds were able to exhibit anticancer effects in a glioma cell line by inducing apoptosis [15]. Our studies confirm that different groups of diterpenes can induce apoptosis through different signaling pathways which is extremely important in the context of their further in vivo studies.

## 4. Materials and Methods

### 4.1. Plant Material

*Plectranthus ornatus* Codd. seeds were provided by the Herbarium at the Lisbon Botanical Garden, Portugal and were cultivated at the Faculty of Pharmacy Hortum at the Lisbon University. Whole plants were collected in May 1998 and voucher specimens were deposited in the Herbarium of the Botanical Center of the “Instituto de Investigacão Científica Tropical,” Lisboa, Portugal (ref. C. Marques S/N_ LISC).

### 4.2. Extraction and Isolation of Compounds

PLEC was isolated as previously described by Rijo et al. (2002) [23] and MRC was isolated as previously described, also by Rijo et al. (2005) [19]. The halimane diterpene, HAL, was isolated from the *P. ornatus* extract using procedures similar to that described by Rijo et al. (2007) [62] in the CBIOS laboratory of Natural Bioactives (Bio.Natural@CBIOS, Universidade Lusófona, Lisboa, Portugal). Briefly, *P. ornatus* (1261 kg) was air-dried and sprayed. An Ultrasound-assisted extraction was performed with acetone three times (13 L total volume) for half an hour at room temperature. The solvent was then filtered and evaporated at 40–50 °C on a rotary evaporator and a dry extract of 89.30 g was obtained, constituting an extraction yield of 7.082% (w/w). The crude extracts were stored in DMSO at 20 mg/mL. The halimane compound was purified by exposing the extract to a combination of sequential liquid and dry column flash chromatography (six total), using a silica-based stationary phase and a mixture of eluents of increasing polarity (*n*-Hexane:Ethyl acetate) as a mobile phase, attaining 5.3 mg of pure compound. The structural elucidation of the metabolites was carried out basing on physicochemical data, including melting point, specific rotation, spectroscopic data (UV, IR, 1D- and 2D- 1H and 13C NMR), mass spectra and elemental analysis, to be compared with data already reported in literature. NMR spectra were recorded on a Bruker Fourier 300 spectrometer, with 1H-NMR and 13C-NMR spectra being recorded at 300 and 75 MHz, respectively. The compounds will be further denoted by their reference name, indicated in Table 1.

### 4.3. Tissue Culture Models

The MCF7 (human breast; mammary gland; adenocarcinoma; HTB-22™) and FaDu (human pharynx squamous cell carcinoma; HTB-43™) cell lines were obtained from the American Type Culture Collection (ATCC; Manassas, VA, USA). The two cell models were cultured in a humidified 5% CO_2_ atmosphere at 37 °C (New Brunswick Galaxy^®®^ 170R CO_2_ Incubator). Monolayer cells were grown in Eagle’s Minimum Essential Medium (EMEM; Sigma-Aldrich) supplemented with 10% fetal bovine serum (Biowest), recommended by a manufacturer. To the cell culture medium was added penicillin (100 U/mL) and streptomycin (100 μg/mL). Then, at 80–85% confluence, the cells were detached and resuspended using TrypLE™ Express Enzyme without phenol red (Gibco™).

### 4.4. Cytotoxicity Assay

The cytotoxicity of MCF7 and FaDu cells was detected by MTT method, consistent with our previous research [17]. In each culture model, the cells were treated with increasing concentrations of HAL, PLEC and MRC (0.39–100 µg/mL). After 24 h of incubation, cell growth was measured. The IC_50_ concentration for HAL and PLEC was used for further studies, while the highest tested concentration was used for MRC. All experiments were carried out in triplicate and performed as at least three independent experiments.

### 4.5. Measurement of ROS Production

Di-chloro-dihydro-fluorescein diacetate assay was used to measure ROS production in MCF7 and FaDu cells after treatment with HAL, PLEC and MRC for 12, 24 and 48 h. The DCFH-DA crosses the cell membrane and enzymatically hydrolyzed by intracellular eterases to non-fluorescent di-chloro-dihydro-fluorescin (DCFH), which is then oxidized to highly fluorescent dichloro-fluorescin (DCF) in the presence of intracellular ROS. Cells were seeded in 96-well black plates at a density of 1×10^5^ cells/well in 50 µL culture medium and allowed to adhere overnight in a CO_2_ incubator at 37 °C. The next day, the cells were incubated for 45 min at 37 °C in presence of 5 µM of DCFH-DA, washed in HBSS and then treated with indicated concentrations of HAL, PLEC and MRC for 12, 24 and 48 h. The readings were taken at 485/20 nm excitation and 528/20 nm emission.

### 4.6. Mitochondrial Superoxide Generation

The production of superoxide by mitochondria was determined using the MitoSOX™ Red reagent. Cells were seeded into black 96-well tissue culture plates with transparent bottom (Greiner) at a density of 1 × 10^5^ cells/well in 100 µL culture medium (without phenol red and FBS) and cultured overnight in a CO_2_ incubator at 37 °C. The next day, MCF-7 and FaDu cells were treated with HAL, PLEC and MRC and incubated for another 24 h under the same conditions. Then after 24 h of incubation, 5 μM MitoSOX Red was added to the cells and incubated further for 30 min at 37 °C in 5% CO_2_ atmosphere (protected from light). Thereafter, the cells were centrifuged (300 g for 5 min at 22 °C) and washed with the HBSS and the fluorescence emission at 590/35 nm under 485/20 nm excitation was measured using a Bio-Tek Synergy HT Microplate Reader. Each sample was run in triplicate.

### 4.7. Examination of MMP

Mitochondrial Membrane Potential was examined by JC-1 fluorescent probe (5′,6,6′-tetrachloro-1,1′,3,3′-tetraethylbenzimidazolylcarbocyanine iodide). JC-1 is a fluorescent carbocyanine dye which accumulates in the mitochondrial membrane in two forms: monomers or dimers, depending on mitochondrial membrane potential. JC-1 monomers show maximum fluorescence excitation and emission at 485 and 538 nm wavelengths, respectively. On the other hand, negative potential of the inner mitochondrial membrane facilitates the formation of dye aggregates, which results in the shift of JC-1 monomer fluorescence (green) towards red light, i.e., excitation at 530 nm and emission at 590 nm wavelengths, respectively. The cells were seeded into black 96-well microplate wells having transparent bottoms (Greiner) at a density of 1×10^5^ cells/well in 50 µL culture medium and kept overnight before treatments for attachment. The next day, cells were treated with indicated concentration of PLEC, HAL and MRC for 24 h. Finally, the cells were pre-incubated with 5 μM JC-1 in the HBSS in a CO_2_ incubator at 37 °C for 30 min. Prior to measurements, the cells were centrifuged (300 g for 10 min at 22 °C) then washed twice with the HBSS. Fluorescence was measured in Bio-Tek Synergy HT Microplate Reader (Bio-Tek Instruments, Winooski, VT, USA) and results are shown as a ratio of fluorescence, measured at 530 nm/590 nm to that measured at 485 nm/538 nm (aggregates to monomer fluorescence). All compounds were tested in triplicate.

### 4.8. DNA Extraction from Cell Culture

The total genomic DNA, including nuclear and mitochondrial, was isolated using the QIAamp DNA Mini Kit according to the manufacturer’s recommendations from treated cell suspensions (2 × 10^6^ cells) collected by centrifugation. The concentration and purity of the isolated DNA was assessed spectrophotometrically.

### 4.9. Determination of mtDNA Copy Number

Quantitative Real-Time PCR (qRT-PCR) was used to examine the relative amounts of mitochondrial DNA (mtDNA) and nuclear DNA (nDNA), as we described earlier [17,63,64,65]. Primers sequences, qRT-PCR conditions are provided in the Supplementary Materials.

### 4.10. Measurements of Mitochondrial and Nuclear DNA Damage

Semi-long run quantitative Real-Time-PCR (SLR-qRT-PCR) was used to estimate mitochondrial DNA (mtDNA) and nuclear DNA (nDNA) damage, according to the protocol described in our previous publications [17,63,64,65]. We have included the primer sequences, SLR-qRT-PCR conditions in the Supplementary Materials.

### 4.11. Measurement of Bax, Bcl-2, Cas-3, Cas-8, Cas-9, Apf-1, MCL-1 and TP53 Gene Expression

For RNA analysis, cells treated for 24 h with PLEC, HAL and MRC were lysed using the ISOLATE II RNA Mini Kit (BIOLINE Ltd.) and next RNA was isolated according to the manufacturer’s instructions. Complementary DNA (cDNA) was generated using the High Capacity cDNA Reverse Transcription Kit (ThermoFisher) and ReverseTranscription-quantitative PCR (RT-qPCR) analysis was performed using the following commercially available TaqMan^®®^ gene expression assays: Bax (Hs00180269_m1), Bcl-2 (Hs00608023_m1), Cas-3(Hs00234387_m1), Cas-8 (Hs01018151_m1), Cas-9 (Hs00962278_m1), Apf-1 (Hs00559441_m1), MCL-1 (Hs01050896_m1), TP53 (Hs00153349_m1) and 18S rRNA (reference gene; Hs99999901_s1). RT-qPCR was performed using TaqMan™ Gene Expression Master Mix (Thermo Fisher Scientific) and Agilent Technologies Stratagene Mx300SP working on MxPro software. The RT-qPCR conditions were as follows: 10 min of polymerase activation at 95 °C, followed by 40 cycles of 30 s denaturation at 95 °C and 60 s annealing/extension at 60 °C. Each sample was run in triplicate. The basal expression level was calculated using the Ct method [66].

### 4.12. Flow Cytometry

Apoptosis was evaluated by flow cytometry with FITC Annexin V Apoptosis Detection Kit I (BD Pharmingen) after treatment with HAL, PLEC and MRC. The cells were washed twice in PBS and then suspended in 100 µL 1 × Binding Buffer at a cell density of 10^5^. A 5 μL FITC Annexin V and 5 μL of propidium iodide (PI) was added to each 100 μL cell suspension, which was then incubated at room temperature (25 °C) for 15 min in the dark. Then after incubation, 400 μL of the 1× Binding Buffer was added to cell suspension and next samples were analyzed by flow cytometry on a LSRII (Becton Dickinson, San Jose, CA, USA) flow cytometer; in total, 5 × 10^4^ cells were analyzed per sample in three independent experiments. Apoptotic index (AI) was calculated as the mean percentage of apoptotic cells (early and late apoptotic cells).

### 4.13. Caspase-3/7 Activity

To determine if HAL, PLEC and MRC promote cell death in MCF-7 and FaDu cell lines we analyzed caspase3/7 activation. After incubation of cell lines with HAL, PLEC and MRC we assessed caspase-3/7 activity by fluorescence microplate reader using the commercial CellEvent™ Caspase-3/7 Green Detection Reagent (Invitrogen™) that is based on the emission of fluorescence upon cleavage of the fluorescently labeled DEVD peptide. We followed the procedure as we described earlier [67,68]. Briefly, MCF-7 and FaDu cells were seeded in 96-well microplate with transparent bottoms at 1×10^5^ cells/well in 50 µL culture medium and incubated at 37 °C, 5% CO_2_ to allow the attachment of cells. After overnight incubation, cells were washed with 1× PBS to remove nonadherent cells, treated with HAL, PLEC and MRC and next incubated for another 24 h under the same conditions. After that time CellEvent Caspase-3/7 Green Detection Reagent was added to the wells at a final concentration of 5 µM. The caspase activity was determined after 30 min of incubation by measuring the fluorescence intensity of cells, at 502 nm excitation wavelength and an emission wavelength of 530 nm, using a Bio-Tek Synergy HT Microplate Reader. Each sample was run in triplicate.

### 4.14. In vivo Assessment of General Toxicity

The overall toxicity of the samples was assessed using the *Artemia salina* lethality bioassay, as previously described [69,70]. Briefly, samples of each compound (HAL, PLEC or MRC) were prepared at a concentration of 100 µg/mL (lowest tested concentration at which a biological effect was observed), with dilutions made in salt medium. Larvae incubated for 24 h at 25 °C were added to each well containing salt medium. After 24 h, the number of dead larvae in each well was recorded and the mortality rate (%) was determined. The study was conducted in triplicate.

### 4.15. Statistical Analyses

All statistical analyses of differences between compounds (HAL, PLEC, MRC) were performed in Prism v. 5.00 for Windows (GraphPadSoftware, Inc., San Diego, CA, USA) using ordinary one-way ANOVA followed with Dunnett’s multiple comparison test. Results were expressed as means with SD. The experiments were performed in triplicate, n ≥ 3. 

## 5. Conclusions

HAL and PLEC demonstrated a cytotoxic effect against MCF7 and FaDu cancer cell lines. In contrast, MRC isolated from *Plectranthus ornatus* indicated weaker biological potential or a different mechanism of action. This data also demonstrated that both compounds triggered the mitochondrion-mediated apoptosis pathway through the production of ROS leading to a change in the expression pro and anti-apoptotic genes (*Bax, Bcl-2, TP53, Cas-3, Casp-8, Casp-9, Apaf-1* and *MCL-1*) followed by a decrease in MMP and mitochondrial copy number, an increase in mitochondrial damage in the ND1 and ND5 gene regions or an increase caspase-3/7 activity. No toxic effect in a simple in vivo test for individual compounds was observed. This result indicates that these compounds may be interesting candidates as potential chemotherapeutic agents; however, further studies are needed to determine the exact mechanism of action and to evaluate their antitumor activity in in vivo.

## Figures and Tables

**Table 1:** Diterpenes isolated from the *Plectranthus ornatus* Codd. and their reference name.

Plant Material	Isolated Compounds	Structure	Reference Name
***Plectranthus ornatus***	(11R*,13E)-11-acetoxyhalima-5,13-dien-15-oic acid	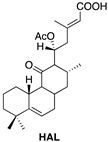	**HAL**
	1α,6β-diacetoxy-8α,13R*-epoxy-14-labden-11-one	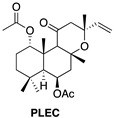	**PLEC**
	1,6-di-*O*-acetylforskolin	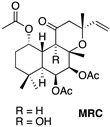	**MRC** **Mixture 1:1**
	1,6-di-*O*-acetyl-9-deoxyforskolin		

## Data Availability

Not applicable.

## Supplementary Materials

The following supporting information can be downloaded at: https://www.mdpi.com/article/10.3390/ijms231911653/s1.
