# Supplementary material for: Anticancer Properties of Plectranthus ornatus-Derived Phytochemicals Inducing Apoptosis via Mitochondrial Pathway
Source: Int J Mol Sci. 2022 Oct 1;23(19):11653. doi: 10.3390/ijms231911653 (PMC9569850; doi:10.3390/ijms231911653)
Supplement: Supplementary file 1 [file ijms-23-11653-s001.zip › ijms-1929312-supplementary.pdf]

Supplementary materials

1. Determination of mtDNA copy number

Table S1. Quantitative real-time PCR primers used in this study.

Gene symbol (gene ID)	Description	Primer sequence (5'→3')	Size (bp)
***ND1* (ID: 4535)**	mitochondrially encoded NADH:ubiquinone oxidoreductase core subunit 1	Forward primer: 5'-CCTAAAACCCGCCACATCTA-3'<br>Reverse primer: 5'-GCCTAGGTTGAGGTTGACCA-3'	124
***ND5* (ID: 4540)**	mitochondrially encoded NADH:ubiquinone oxidoreductase core subunit 5	Forward primer: 5'-AGGCGCTATCACCACTCTGT-3'<br>Reverse primer: 5'-TTGGTTGATGCCGATTGTAA-3'	124
***SLCO2B1* (ID: 11309)**	solute carrier organic anion transporter family member 2B1	Forward primer: 5'-TGCAGCTTCCTCTTCACAGA-3'<br>Reverse primer: 5'-CTCAGCCCCAAGTATCTCCA-3'	135
***SERPINA1* (ID: 5265)**	serpin family A member 1	Forward primer: 5'-GATCCCAGCCAGTGGACTTA-3'<br>Reverse primer: 5'-CCTGAAGCTGAGGAGACAGG-3'	148

<u>RT-qPCR conditions:</u>

Step 1 at 95 ºC for 3 min;

Step 2 at 95 ºC for 15 sec;

Step 3 at 65 ºC for 30 sec;

Step 5 at 72 ºC for 15 sec; with the plate reading:

Repeat steps 2 × 39 cycles

2. Measurements of mitochondrial and nuclear DNA damage

<u>RT-qPCR conditions:</u>

Step 1 at 95 ºC for 3 min;

Step 2 at 95 ºC for 15 sec;

Step 3 at 65 ºC for 30 sec;

Step 5 at 72 ºC for 15 sec (short fragments) or 45 s at 72°C (long fragments); with the plate reading:

Repeat steps 2 × 39 cycles

Table S2. Description of semi-long run RT-PCR primers used for mitochondrial and nuclear DNA damage quantification.

Genome	Target gene	Forward primer sequences (5'→3')	Reverse primer sequence (5'→3')	Amplicon length (bp)
**Mitochondrial**	*ND1* (mitochondrially encoded NADH:ubiquinone oxidoreductase core subunit 1)	Long fragment: ATGGCCAACCTCCTACTCCT	Long fragment: GATGAGTGTGCCTGCAAAGA	1214
		Small fragment: CCTAAAACCCGCCACATCTA	Small fragment: GCCTAGGTTGAGGTTGACCA	124
	ND5 (mitochondrially encoded NADH:ubiquinone oxidoreductase core subunit 5)	Long fragment: TCCAACTCATGAGACCCACA	Long fragment: AGGTGATGATGGAGGTGGAG	1156
		Small fragment: AGGCGCTATCACCACTCTGT	Small fragment: TTGGTTGATGCCGATTGTAA	124
**Nuclear**	*TP53* (tumor protein p53)	Long fragment: GGGTGTAGATGATGGGGATG	Long fragment: AACTGCGGAATGAAACAACC	1172
		Small fragment: AAGCTGCTAAGGTCCCACAA	Small fragment: GGAAAGATCGCTCCAGGAA	56
	HPRT1 (hypoxanthine phosphoribosyltransferase 1)	Long fragment: AGGGCAAAGGATGTGTTACG	Long fragment: AGTGGTTTCTGGTGCGACTT	1018
		Small fragment: TGCTGACCTGCTGGATTACA	Small fragment: TCTACAGTCATAGGAATGGATCTATCA	69
